# In Vitro Determination of the Immunogenic Impact of Nanomaterials on Primary Peripheral Blood Mononuclear Cells

**DOI:** 10.3390/ijms21165610

**Published:** 2020-08-05

**Authors:** Christopher A. W. David, Michael Barrow, Patricia Murray, Matthew J. Rosseinsky, Andrew Owen, Neill J. Liptrott

**Affiliations:** 1 Department of Molecular and Clinical Pharmacology, Institute of Translational Medicine, University of Liverpool, Liverpool L69 3BX, UK; cdavid@liverpool.ac.uk (C.A.W.D.); aowen@liverpool.ac.uk (A.O.); 2 Department of Chemistry, University of Liverpool, Liverpool L69 7ZD, UK; m.barrow@liverpool.ac.uk (M.B.); rossein@liverpool.ac.uk (M.J.R.); 3 Department of Cellular and Molecular Physiology, University of Liverpool, Liverpool L69 3BX, UK; embryo@liverpool.ac.uk; 4 Centre for Preclinical Imaging, University of Liverpool, Liverpool L69 3BX, UK

**Keywords:** nanomaterials, nanoparticles, nanotoxicology, immunotoxicology, inflammasome

## Abstract

Investigation of the potential for nanomaterials to generate immunogenic effects is a key aspect of a robust preclinical evaluation. In combination with physicochemical characterization, such assessments also provide context for how material attributes influence biological outcomes. Furthermore, appropriate models for these assessments allow accurate in vitro to in vivo extrapolation, which is vital for the mechanistic understanding of nanomaterial action. Here we have assessed the immunogenic impact of a small panel of commercially available and in-house prepared nanomaterials on primary human peripheral blood mononuclear cells (PBMCs). A diethylaminoethyl-dextran (DEAE-dex) functionalized superparamagnetic iron oxide nanoparticle (SPION) generated detectable quantities of tumor necrosis factor α (TNFα), interleukin-1β (IL-1β), and IL-10, the only tested material to do so. The human leukemia monocytic cell line THP-1 was used to assess the potential for the nanomaterial panel to affect cellular oxidation-reduction (REDOX) via measurement of reactive oxygen species and reduced glutathione. Negatively charged sulfonate-functionalized polystyrene nanoparticles demonstrated a size-related trend for the inhibition of caspase-1, which was not observed for amine-functionalized polystyrene of similar sizes. Silica nanoparticles (310 nm) resulted in a 93% increase in proliferation compared to the untreated control (*p* < 0.01). No other nanomaterial treatments resulted in significant change from that of unstimulated PBMCs. Responses to the nanomaterials in the assays described demonstrate the utility of primary cells as ex vivo models for nanomaterial biological impact.

## 1. Introduction

The ideal design of a preclinical assessment is a simple, reliable, robust methodology with good in vitro to in vivo correlation. Determination of immunotoxicity potential is a vital part of translation for nanomedicines and other materials [1,2]. The complex nature of nanoparticles in terms of their physicochemical characteristics lends to the need for rigorous investigation to establish those that may contribute to adverse immune effects [3]. In order to perform this on a viable scale, higher throughput assays are needed. As such, the development of in vitro assays, which are able to generate translatable results to support further in vivo testing, is favourable in terms of de-risking development and in terms of minimising the need for animal sacrifice in line with the 3Rs principles of replace, reduce, and refine. In order to achieve this, the use of primary cells in preclinical in vitro analysis is becoming more widespread.

Thorough assessment of the potential modulation of cytokine profiles by new drugs and therapies has been highlighted by the outcomes of the phase 1 clinical trial of theralizumab, an anti-CD28 monoclonal antibody. Six volunteers received a 0.1 mg dose of theralizumab per kilogram of body weight [4], 500-times smaller than that deemed safe by preclinical animal studies [5]. Following infusion, a series of adverse effects involving multiple organ failure were elicited by unexpected cytokine release [5]. The “cytokine storm”, a term coined by Aikawa [6], is the elevation of both proinflammatory and anti-inflammatory cytokines leading to the deleterious effects observed in the trial. It is important to note that the systemic inflammatory response generated by theralizumab was in the absence of contaminating organic factors [7]. The cytokine storm to this drug was not observed in the preclinical studies performed in rats and cynomolgus monkeys [4], but would have been detected using an in vitro cytokine release assay in primary human blood [8].

Cytokines as biomarkers of nanoparticle immunomodulatory properties demonstrate good correlation to that to that observed in vivo [3,9]. Four cytokines, namely interferon γ (IFNγ), TNFα, IL-1β, and IL-10, were chosen for assessment. This selection was based on the inter-related roles these cytokines play, and allowing observation of the influence that nanoparticles have on the generation of these inflammatory (TNFα, IL-1β), anti-inflammatory (IL-10), and pleotropic (IFNγ) cytokines.

The generation of reactive oxygen species (ROS) has been associated with numerous nanomaterials as a mechanism of nanoparticle-mediated toxicity [10]. This has been found as the result of the induction of proinflammatory factors, or processes including the generation of free radicals via Fenton-type reactions [11], mitochondrial localization [12], and mitochondrial dysfunction via other mechanisms [13]. Mitochondrial ROS is well-known to promote proinflammatory cytokine production [14], and has been demonstrated to have a proliferative effect at certain levels [15]. Cell type-specific interactions have also been shown, such as frustrated phagocytosis [16], which has been associated with carbon nanotubes [13].

Nanoparticles, including silica and titanium dioxide, have been shown to interact with inflammasomes [17] which are involved in the maturation of the pro-inflammatory cytokines IL-1β and IL-18 [18]. NLR family pyrin domain containing 3 (NLRP3) inflammasome activation by endogenous and exogenous danger signals leads to the release of mature IL-1β via the action of caspase-1 [19]. The activation of caspase-1 is known to be the rate-limiting step in inflammation due to IL-1β or IL-18 [20]. Being such a key component in the regulation and generation of IL-1β, the potential for nanoparticle interference is important.

The proliferative response of leukocytes to stimulators and cytokine production is vital in the normal generation of an immune response. The utility of assessing this process in vitro can evaluate the potential of nanomaterials for being immunostimulatory or immunosuppressive [3]. The latter involves the use of a known mitogen/antigen to determine the extent of the potential suppression of proliferation. A correlation was previously shown between in vitro and in vivo murine mitogen responses and subsequent immunosuppression by nanomaterials [21].

The aim of the work presented here was to evaluate cytokine secretion by PBMCs in response to nanoparticles. This was performed by direct quantification, as well as observing the influence of nanomaterials on caspase-1 activity, and inflammasome activation. The impact on proliferation of primary human leukocytes was also assessed.

## 2. Results

### 2.1. Secretion of Cytokines from Healthy Volunteer PBMCs Following 24-h Exposure to Nanomaterials

No materials generated an IFNγ response at a level greater than the lower limit of detection for the assay (6.93 pg/mL). Furthermore, the levels of TNFα, IL-1β, and IL-10 were below the detectable limit of the assay (15.40 pg/mL, 3.85 pg/mL, 8.42 pg/mL, respectively) in untreated controls precluding the calculation of fold difference and formal statistical analysis.

**Figure 1:**
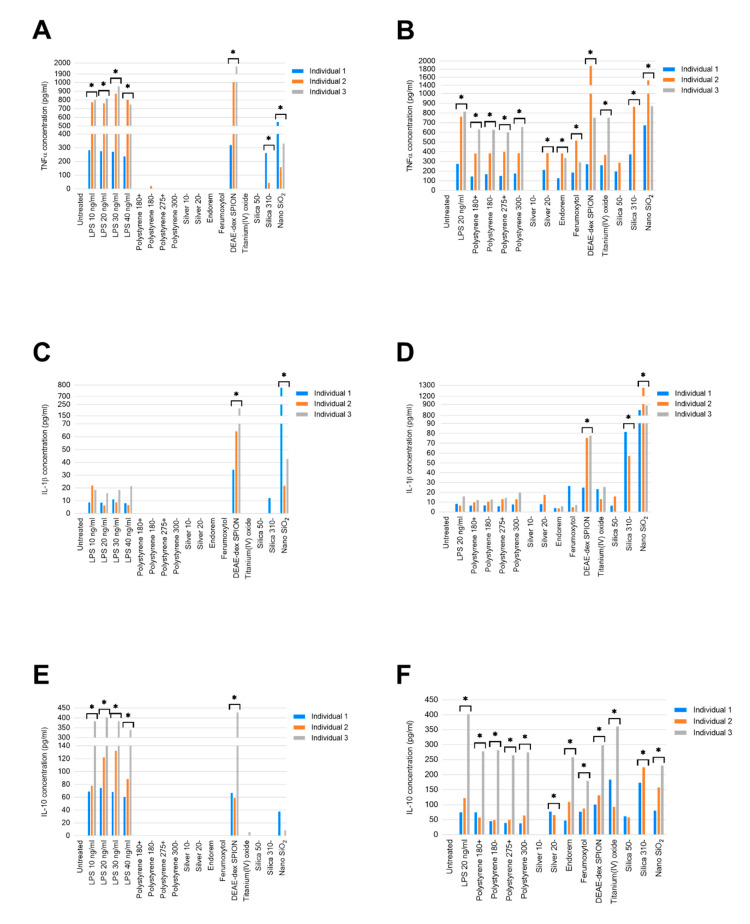
Concentrations of stated cytokines secreted by peripheral blood mononuclear cells in response to (**A**,**C**,**E**) treatment with LPS, or stated nanomaterials; (**B**,**D**,**F**) Combined LPS treatment with stated nanomaterials. Data displayed as an average of two technical replicates. * *p*-value < 0.05.

Detectable concentrations of TNFα were generated in response to all tested concentrations of lipopolysaccharide (LPS) for all three individuals (Figure 1A). The concentrations of TNFα in LPS treatments demonstrated a high degree of similarity in individuals 2 and 3. The concentrations generated in PBMCs from individual 1 were approximately 2.5-times lower at all LPS concentrations, an interindividual variability normal for immunology assays. DEAE-dex SPION and nano SiO_2_ generated significant TNFα in comparison to untreated PBMCs from all three individuals (*p* ≤ 0.0001). Treatment with silica 310− resulted in TNFα generation in PBMCs from individuals 1 (261.95 pg/mL) and 2 (44.35 pg/mL), but not 3. Only individual 2 was found to have produced TNFα in response to polystyrene 180− (21.86 pg/mL).

Consistent reductions of TNFα concentrations (~40%, ~50%, and ~25% within individuals 1, 2, and 3, respectively) were observed in combined treatments with LPS and all polystyrene nanoparticles compared to positive controls (Figure 1B). Endorem- and ferumoxytol-combined LPS treatments also resulted in lower concentrations of TNFα than solely LPS (20 ng/mL) treated PBMCs. However, combined LPS and silica 310− and nano SiO_2_ resulted in higher concentrations of TNFα in samples from individuals 1 and 2.

Treatment with LPS at all tested concentrations, DEAE-dex SPION, and nano SiO_2_ resulted in detectable quantities of IL-1β from individuals 1, 2, and 3. The most marked of these was that of individual 1 in response to treatment with 100 µg/mL of nano SiO_2_, which was 18-times greater than individual 3, and 36-times greater than individual 1 (Figure 1C). Nano SiO_2_ generated an IL-1β concentration of 11.99 pg/mL in the sample from individual 1, the only one to do so in response to this nanomaterial.

Combined treatment with LPS and nano SiO_2_ resulted in the highest concentration of IL-1β generated in response to all tested conditions in PBMCs from all individuals (*p* ≤ 0.0001, Figure 1D).

LPS treatment across all concentrations, and DEAE-dex SPION, resulted in the secretion of IL-10 in PBMCs from all individuals (*p* ≤ 0.0001), the most pronounced effect being observed from individual 3 (Figure 1E). Nano SiO_2_ was found to have stimulated IL-10 secretion in individuals 1 (37.84 pg/mL) and 3 (8.69 pg/mL), while only individual 3 produced a quantifiable concentration of this cytokine in response to titanium(IV) oxide (5.85 pg/mL).

All LPS-combined nanomaterial treatments resulted in less IL-10 produced than treatment solely with 20 ng/mL LPS in PBMCs from individual 3 (Figure 1F). Combined treatments of silver 20−, ferumoxytol, titanium(IV) oxide, silica 310− and nano SiO_2_ with LPS generated more IL-10 than LPS-only treatments by individual 1. This increased production was observed in individual 2 from LPS with DEAE-dex SPION, silica 310−, and nano SiO_2_. Combined treatment with LPS and silver 10− resulted in undetectable concentrations of TNFα, IL-1β and IL-10 in all individuals.

### 2.2. Nanomaterial Modulation of THP-1 REDOX

**Figure 2:**
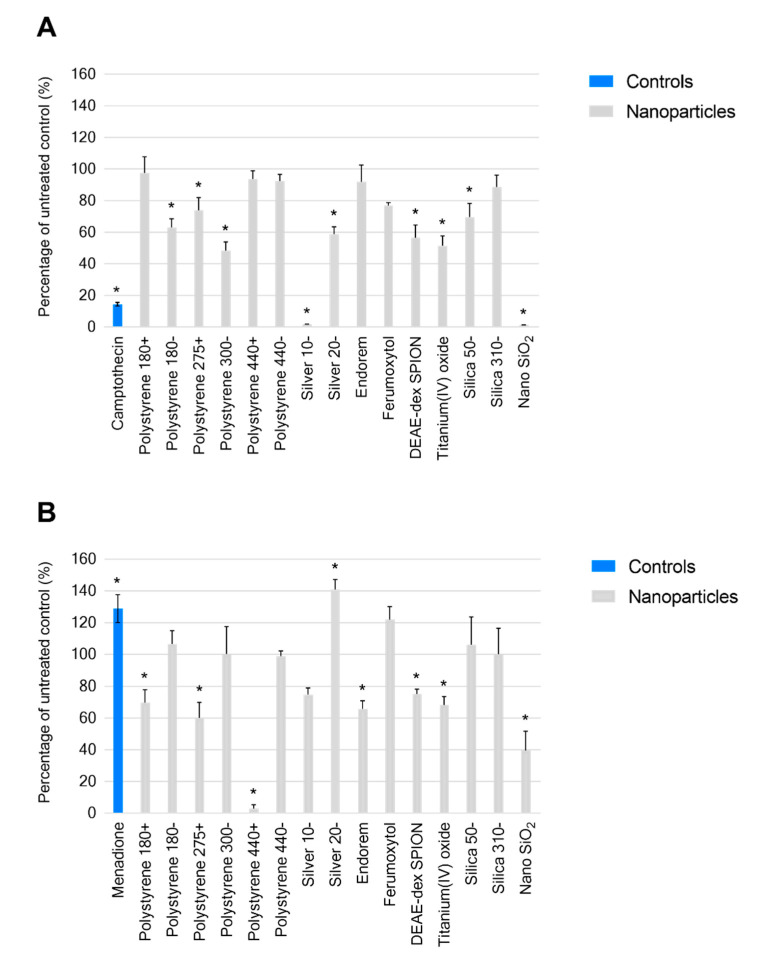
(**A**) Generation of reactive oxygen species, and (**B**) reduced glutathione in the presence of stated nanomaterials as a percentage of untreated of control. Data displayed as an average of four technical replicates ± standard deviation. * *p*-value < 0.05.

Treatment with the positive control camptothecin, a known inducer of reactive oxygen species, for 24 hours resulted in an observed level of ROS that was 86% lower than that of the untreated control (Figure 2A). Silver 10− resulted in a level of ROS 98.4% (*p* ≤ 0.0001) less than the untreated control at a concentration of 100 µg/mL, and nano SiO_2_, a material demonstrating no known physicochemical similarity, led to a similar observation at the same concentration (98.8% less, *p* ≤ 0.0001). A significant but not as pronounced effect was observed from silver 20− and silicas 50−.

At the same timepoint, treatment with the positive control menadione resulted in 29% (*p* ≤ 0.0001) more reduced glutathione than the untreated control (Figure 2B). Nano SiO_2_ led to a level 60.5% (*p* ≤ 0.0001) lower than the control, whereas treatment with silicas 50− and 310− did not affect the level of reduced glutathione under the described conditions at the tested concentration (100 µg/mL). Silver 10− did not result in significant change, while treatment with silver 20− led to 41% (<0.0001) more reduced glutathione. The most pronounced observed impact was by polystyrene 440+ (97% less, *p* ≤ 0.0001), and significant effects were also generated by polystyrene 275+ (39.8% less, *p* ≤ 0.0001), and polystyrene 180+ (30.2% less, *p* = 0.01).

### 2.3. Cell-Free Determination of Influence on Caspase-1

**Figure 3:**
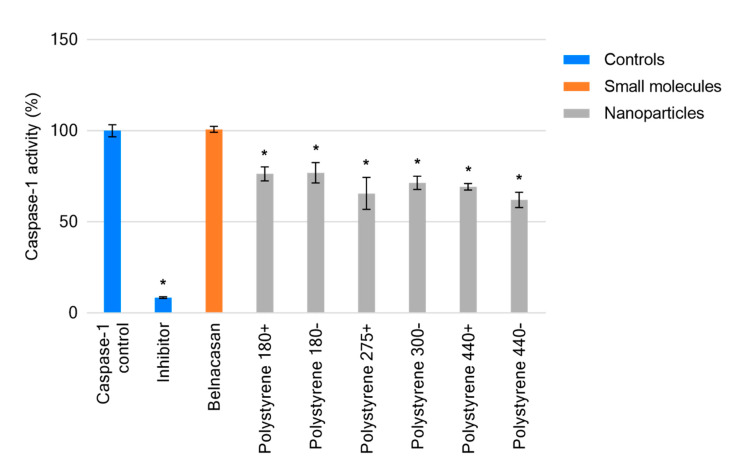
Caspase-1 activity displayed as a percentage of total activity in the caspase-1 control, following treatments with small molecules and stated nanomaterials. Data displayed as an average of three technical replicates ± standard deviation. * *p*-value < 0.05.

The caspase-1 inhibitor carbobenzoxy-valyl-alanyl-aspartyl-[*O*-methyl]- fluoromethylketone (Z-VAD-FMK), provided with the kit as a positive control, reduced caspase-1 function by 92.5% (*p* < 0.0001). All treatments demonstrated significant inhibition of caspase-1 (*p* < 0.0001) with the exception of belnacasan, which had no effect on caspase-1 activity compared to the untreated control. Negatively charged sulfonate-functionalized polystyrenes 180−, 300−, and 440− demonstrated increasing inhibition of caspase-1 (23%, 28.6%, and 38%) consistent with their increasing size. However, this trend was absent in quaternary amine-functionalized polystyrenes 180+, 275+, and 440+ (Figure 3). Of these materials, the greatest effect was demonstrated by polystyrene 275+ (275 nm manufacturer provided size) inhibiting caspase-1 by 34.4%.

### 2.4. Leukocyte Proliferation Following 48-h Exposure to Nanomaterials

**Figure 4:**
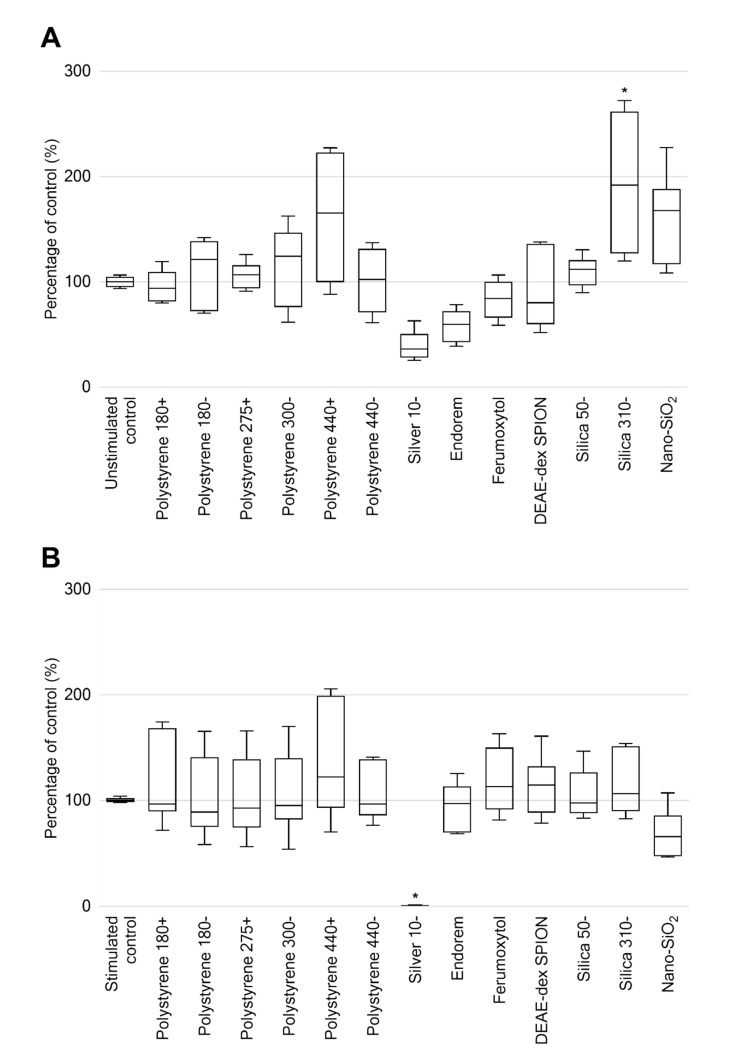
Proliferation of peripheral blood mononuclear cells, as a percentage of controls, following treatment with (**A**) stated nanomaterials. (**B**) Combined treatment with PHA and stated nanomaterials. Data displayed as box and whisker plots showing average of six technical replicates, maximum, and minimum ± standard deviation. * *p*-value < 0.05.

Silica 310−, the largest sized silica nanoparticle at 310 nm (manufacturer’s provided size), resulted in a 93% increase in proliferation compared to the untreated control (*p* < 0.01). No other nanomaterial treatments resulted in significant change from that of the unstimulated control (Figure 4A). A significant change in proliferation of phytohemagglutinin-M (PHA) stimulated nanomaterial treatments was only observed for silver 10−, where no viable cellular presence was found (*p* < 0.0001). No other nanomaterial treatments of PHA-stimulated PBMCs resulted in significant change from that of the PHA-stimulated control (Figure 4B).

## 3. Discussion

The need for thorough investigation of the immunological consequences of medicines in human model systems is dramatically exemplified by the case of theralizumab. This study sought to assess the utility of primary human cells as ex vivo models for the biological impact of nanomaterials. Peripheral blood mononuclear cells isolated from healthy donor blood were challenged with a small panel of commercially available and in-house prepared nanomaterials to observe their potential immunogenic impact.

The interplay of cytokines in vivo represents a highly complex multicomponent system in which the secretion of cytokines from various cells can generate, suppress, or recruit further cell types to the site of inflammation. Dysregulation of cytokine balance is a hallmark of numerous disease states [22], but also a source of opportunity for exploitation in the rational design of nanomedicines for immunomodulatory medicines [23].

IL-1β is a marker of the pyrogenic response and the intentional design of materials to stimulate its production in vivo is of great interest for vaccine development [24]. Iron oxide nanoparticles have been demonstrated to have great potential in this area. Pusic et al. have described that murine dendritic cells treated with <20 nm carboxyl-functionalized nanoparticles resulted in the generation of a range of cytokines including IL-1β, TNFα, and IFNγ [25]. Ferumoxytol, a 30 nm carboxyl-functionalized iron oxide nanoparticle, was not found to generate the secretion of any tested cytokines under the experimental conditions described in this work. Ferumoxytol possesses a negative zeta potential. While it can be assumed that the iron oxide nanoparticles assessed by Pusic et al. were also of negative charge, a qualitative determination of charge by gel electrophoresis does not provide sufficient evidence for comparison. The only iron oxide nanoparticle from the chosen panel presented here that resulted in an increased production of cytokines, without LPS stimulation, was DEAE-dex SPION (diethylaminoethyl dextran coated, 52.3 nm hydrodynamic size). Similar to the observations in the study by Pusic et al., DEAE-dex SPION was found to increase IL-1β and TNFα compared to an untreated control. Unlike the described study, IFNγ was not generated to a concentration detectable via the multiplex methodology used (>6.93 pg/mL). This is important to highlight the potential biological difference between outwardly similar nanoparticles.

TNFα, IL-1β, and IL-10 are known to be stimulated by LPS in PBMCs [26]. Our data supports this observation while also confirming the lack of influence over IFNγ. In order to thoroughly assess the potential inhibitory effects of nanomaterials on the type II interferon IFNγ, use of a different stimulator would be invaluable. PHA is a known IFNγ stimulator [27], in addition to IL-2, and granulocyte-macrophage colony-stimulating factor (GM-CSF) [28]. In this work, PHA was utilized for its action as a mitogen. When comparing the mitogenic potential of PHA to that of tested nanomaterials, it must be noted that its rate and mode of action is different, involving binding to T cell membranes [29]. Additionally, PHA increases membrane permeability [30] and while it is commonplace, co-incubation to assess nanoparticle-related inhibition of proliferation [27] may alter the rate and/or mechanism of nanoparticle uptake compared to that in cells not stimulated with PHA. No literature has been found to investigate the potential for this effect in comparison to other mitogenic compounds.

NLRP3 inflammasome induction is indicated by the secretion of IL-1β [19]. Silica nanoparticles have been associated with the activation of the NLRP3 inflammasome [31,32,33]. Treatment of LPS-primed PBMCs with nano SiO_2_ was found to generate IL-1β concentrations much higher than any other treatment in all individuals. It has been shown previously that sub-micron sized silica particles induce higher levels of IL-1β production as a result of lysosomal dysfunction than those of larger sizes [34]. Nano SiO_2_ is known to be the largest of the three tested silica nanoparticles (1062.60 nm). The contradictory result obtained here compared to that described by Kusaka et al. implies that size is not the only determinant. All three silica nanoparticles displayed near-identical zeta potential in Roswell Park Memorial Institute (RPMI) 1640 medium supplemented 10% with fetal bovine serum (FBS); −8.78, −8.53, and −8.60, respectively. Silicas 50− and 310− are known to be stabilzsed with L-arginine, but the stabilization applied to nano SiO_2_ is unknown.

The increasing human exposure to nanomaterials creates cause for concern with regard to their potential to modulate and affect cellular health. The route of exposure should be a primary consideration for a well-informed, mechanistic, approached assessment [35]. To observe the interaction of nanomaterials with cells in a manner which may be extrapolated to an in vivo response, a mixed cell population shows utility. This is exemplified by the use of cytokine secretion as an endpoint in this work where, for example, monocytes will contribute primarily IL-1β/TNFα while T cells generate IL-10 [36]. The investigation of other nano-bio interactions may be confounded by mixed populations, and the use of a homogenous model may demonstrate greater utility. THP-1 as a model of monocytes represents an abundant cellular subset that nanoparticles will encounter in the bloodstream, comprising between 10% and 30% of peripheral blood mononuclear cells in healthy individuals. THP-1 are a widely utilized in vitro model in the literature, and have been validated in their use by numerous sources [37,38,39]. Amino-functionalized polystyrene nanoparticles have been found to generate ROS in primary human macrophages, which leads to activation of caspase-1, inducing IL-1β [40]. Amine-functionalized polystyrene nanoparticles also induced cell death, increased oxidative stress, mitochondrial disruption and release of cytochrome C, indicating apoptotic cell death in primary human alveolar macrophages [41]. Polystyrenes 180+, 275+, and 440+ possess the most similar surface-functionalization, however they did not display any of these effects under the described conditions.

Direct quantification of reactive oxygen species poses inherent difficulty as the presence of these radicals is short-lived (approximately 1 × 10^−9^ s) prior to their reaction with cellular components [10]. Investigation of the level of glutathione, due to its rate limited enzymatic conversion [42], provides an alternative measure for oxidative stress, possessing a greater window for observation. Iron oxide nanoparticles have been implicated with depletion of glutathione by a number of sources [43,44]. Endorem and DEAE-dex SPION demonstrated such an effect at a concentration of 100 µg/mL. Reduced glutathione was higher than the untreated control in all tested concentrations of ferumoxytol.

Belnacasan is a prodrug requiring conversion by plasma and liver esterases to the active O-desethyl-belnacasan, a potent caspase-1 inhibitor [45]. The lack of enzymatic conversion validates the absence of observable caspase-1 activity in the chosen cell-free assay.

Lunov et al. have described 100 nm amino-functionalized polystyrene nanoparticles, but not carboxy- or non-functionalized of the same size, to activate caspase-1 through reactive oxygen species generation following lysosomal destabilization [40]. The quaternary ammonium-(polystyrenes 180+, 275+, 440+) and sulfonate-(polystyrenes 180−, 300−, 440−) functionalized polystyrene nanoparticles of varying sizes assessed in this work demonstrated a highly similar inhibitory effect toward caspase-1. The cell-free nature of the assay design shows that inhibition of caspase-1 by polystyrene nanoparticles is a direct interaction of the material, and not the result of further nanoparticle-related effects. This too may be the case but would require further investigation. By utilizing this methodology, it can be elucidated whether the inhibition of caspase-1 is a result of nanomaterials directly on caspase-1, or whether there is an interaction with the mechanisms required for its generation.

In addition to consistency with examples in the literature [46], the decision to utilize the uptake of radioactive thymidine over other colorimetric methodologies was to negate any potential interference that nanomaterials may pose on the assays without excess wash steps. Being able to assess the potential recruitment of other cell types, which may modulate an inflammatory response to nanoparticles in vivo, would be of great advantage in current and future perspectives in order to extrapolate in vitro observations to in vivo outcomes. As mentioned previously, the ability to assess new nanomaterials in a high-throughput manner is of great interest not only to the scientific community, but also to those developing these materials with the intent to bring them to commercial market by reducing potential bottlenecks which occur as a result of lengthy assessment procedures.

The use of freshly isolated primary cells provides the most accurate representation of the in vivo response that may be generated toward nanoparticles. Such an advantage does exist parallel to the main limitation of using such a strategy, namely interindividual variability [47], as demonstrated by the data presented in this work. Although often described as a limitation, this variability is important to try to identify specific interactions in individuals. The interindividual variability of PBMC cultures is known to be higher than that of whole blood [28]. The authors also point out that the potential for cytokine production is highest in whole blood or PBMC cultures where the cell density matches that of whole blood. While simulating such physiological conditions may be the ideal scenario, it lends further technical complications in vitro, and when considering working with precious nanomaterials where only limited quantities are available. This kind of assay design would be best suited to materials that have a known in vivo dosage. However, this information is not readily available for nanomaterials that are not intended for clinical use, or unloaded nanocarriers whose dosage would be determined by the loading efficiency of the associated drug.

It is well known that factors including the time of sampling (chrono-immunology) [48], age [49], gender [50], diet [51], medications and pre-existing conditions all affect the immune system. These, in turn, may modulate the manner and magnitude of any immune responses toward challenge by nanomaterials. The alternative, without performing a large-scale clinical study in which all environmental factors can be observed and potentially controlled, is to perform immunophenotypic (and potentially genotypic) analysis on donors in addition to the assays of interest. The advantages of this would not only be limited to the interpretation of data generated, but in the subsequent dissemination of results to the scientific community.

There are already calls from researchers and governing bodies for minimum reports of physicochemical details and experimental conditions [52]. The current climate of broad conclusions being drawn over variables such as nanoparticle size and charge from extremely limited exemplars being tested is highly detrimental to the development of the field. Further, demographics of the sources of primary samples can only aid in the efforts to create truly transferable datasets [53]. Efforts to catalogue existing information to databases, such as caNanoLab (https://cananolab.nci.nih.gov/caNanoLab/), exemplify the need for standardization in the presentation of information surrounding generated data, which is commonly excluded in order to comply with the conventions of publication. While this is necessary in the current scientific climate, the outlook for high-powered computation of the associations between physicochemical characteristics and immunological effects requires these considerations to be taken sooner rather than later.

Responses to the nanomaterials in the assays described here demonstrate the utility of primary cells as ex vivo models to assess nanomaterial biological impact. The data generated herein serves to support the need for robust assessment methodologies and the need for greater characterization, both of physicochemical characteristics and model systems, to gain insight into the biological outcomes.

## 4. Materials and Methods 

### 4.1. Materials

DEAE-dex SPION was prepared in-house (University of Liverpool, Liverpool, UK) as described by Sharkey et al. [54]. Endorem was purchased from Guerbet (Guerbet GmbH, Sulzbach, Germany), Ferumoxytol was kindly provided for research by AMAG Pharmaceuticals (AMAG Pharmaceuticals, Waltham, MA, USA). Quaternary ammonium functionalized polystyrene ((180+) 180 nm, (275+) 275 nm, (440+) 440 nm), sulfonate functionalized polystyrene ((180−) 180 nm, (300−) 300 nm, (440−) 440 nm), L-arginine stabilized silica ((50−) 50 nm, (310−) 310 nm) and sodium polyacrylate stabilized silver ((10−) 10 nm) were purchased from Sciventions (Sciventions, Toronto, ON, Canada). Nano SiO_2_, LPS, and belnacasan were purchased from Invivogen (InvivoGen, San Diego, CA, USA). Sodium citrate stabilized silver ((20−) 20 nm), titanium(IV) oxide, camptothecin, menadione, RPMI-1640, FBS, Hanks’ balanced salt solution (HBSS), penicillin–streptomycin, heat inactivated human AB serum, 4-(2-hydroxyethyl)-1-piperazineethanesulfonic acid (HEPES) solution, dimethyl sulfoxide (DMSO), L-glutamine solution, PHA, and transferrin were purchased from Sigma-Aldrich (Dorset, UK). CellROX Green Reagent and ThiolTracker Violet were purchased from Thermo Fisher (Cheshire, UK). MACSQuant running buffer was purchased from Miltenyi Biotec GmbH (Bergisch Gladbach, Germany). Ficoll-Paque was purchased from Fisher Scientific (Loughborough, UK). Bio-Plex Pro reagent kit containing antibody-coupled detection beads for human IFNγ, TNFα, IL-1β, IL-10 was purchased from Bio-Rad Laboratories (Hemel Hempstead, UK). The Caspase-1 Inhibitor Drug Detection Kit was purchased from ABCAM (Cambridge, UK).

### 4.2. Methods

#### 4.2.1. Peripheral Blood Mononuclear Cell Isolation

Healthy volunteer blood was collected from the National Blood Service (Liverpool, UK) or from healthy volunteers. For the latter, blood was collected via venipuncture under ethical approval granted by the University of Liverpool Committee in Research Ethics (Ref: RETH000563, approved 1 June 2012). Informed consent given by the volunteers for the use of whole blood in subsequent assays. All samples were anonymized.

Blood was layered over Ficoll-Paque separation medium at a 2:1 ratio and centrifuged for 30 min at 2000 rpm without brake. The PBMC layer was transferred to a fresh universal tube and washed three times in HBSS. PBMCs were suspended in RPMI-1640 supplemented 10% with FBS. PBMC were then counted and resuspended to the required density for subsequent experiments. 

Cells which comprise PBMCs include 70–90% lymphocytes (T cells, B cells, and NK cells), 10–20% monocytes, and 1–2% dendritic cells with some interindividual variability in frequency [55].

#### 4.2.2. Secretion of Cytokines from Healthy Volunteer PBMC Following 24-h Exposure to Nanomaterials

PBMCs were seeded at a density of 2.5 × 10^5^ per well in 1 mL of culture medium in 48-well microplates. Conditions consisted of untreated PBMC, LPS (10, 20, 30, or 40 ng/mL), polystyrene 180+ (100 µg/mL), polystyrene 180− (100 µg/mL), polystyrene 275+ (100 µg/mL), polystyrene 300− (100 µg/mL), silver 10− (100 µg/mL), silver 20− (1 µg/mL), endorem (10 µg/mL), ferumoxytol (10 µg/mL), DEAE-dex SPION (1 µg/mL), titanium(IV) oxide (100 µg/mL), silica 50− (100 µg/mL), silica 310− (100 µg/mL), nano SiO_2_ (100 µg/mL), or combined treatments of nanomaterials at stated concentrations with 20 ng/mL of LPS. Cultures were incubated for 24 h at 37 °C and 5% CO_2_. Aliquots of culture supernatant fractions (100 µL) were taken for analysis of cytokine secretion. Determination of cytokine concentrations was carried out using multiplex cytokine assays conducted using the Bio-Plex 200 Luminex system (Bio-Rad). IFNγ, TNFα, IL-1β, and IL-10 were measured. Briefly, coupled beads (50 µL) were added to every well of a 96-well plate. The plate was washed with wash buffer three times using an automated magnetic plate washer prior to cell culture supernatant fractions (50 µL) being added to the plate, alongside multiplexed standard curves for relevant cytokines. Samples were incubated on a plate shaker for 30 min at room temperature. The plate was then washed three times prior to the addition of detection antibodies (25 µL) and then incubated on a plate shaker for 30 min at room temperature. The plate was again washed three times prior to the addition of streptavidin-phycoerythrin (PE) antibodies (50 µL) and incubation on a plate shaker for 10 min. The plate was then washed three times and assay buffer (125 µL) added to wells. The plate was analyzed on a Bioplex 200 analyzer using recommended gating settings.

#### 4.2.3. Impact on THP-1 ROS generation following 24-h exposure to nanomaterials

THP-1 cells were seeded at 5 × 10^5^ per well in 100 µl of culture medium in 96-well microplates. Conditions consisted of untreated control, polystyrene 180+ (100 µg/mL), polystyrene 180− (100 µg/mL), polystyrene 275+ (100 µg/mL), polystyrene 300− (100 µg/mL), polystyrene 440+ (100 µg/mL), polystyrene 440− (100 µg/mL), silver 10− (100 µg/mL), silver 20− (1 µg/mL), endorem (10 µg/mL), ferumoxytol (10 µg/mL), DEAE-dex SPION (1 µg/mL), titanium(IV) oxide (100 µg/mL), silica 50− (100 µg/mL), silica 310− (100 µg/mL), nano SiO_2_ (100 µg/mL) and positive control camptothecin (10 µM). Following 24 h incubation (37 °C, 5% CO_2_), staining was performed using CellROX Green. The probe was added to all wells (with the exception of background untreated cells) at a final concentration of 5 µM, and incubated for 30 min (37 °C, 5% CO_2_). The plate was centrifuged at 2000 rpm for 5 min, and the supernatant was removed. Cells were washed in 100 µL HBSS followed by centrifugation, repeated three times. Following the final aspiration, cells were suspended in 100 µL of MACSQuant running buffer (Miltenyi Biotec) and transferred to a deep well 96-well microplate. Quantification was performed by flow cytometry (MACSQuant, Miltenyi Biotec) using the fluorescein isothiocyanate (FITC) channel.

#### 4.2.4. Impact on THP-1 Reduced Glutathione Following 24-h Exposure to Nanomaterials

THP-1 cells were seeded at 5 × 10^5^ per well in 100 µL of culture medium in 96-well microplates. Conditions consisted of an untreated control, polystyrene 180+ (100 µg/mL), polystyrene 180− (100 µg/mL), polystyrene 275+ (100 µg/mL), polystyrene 300− (100 µg/mL), polystyrene 440+ (100 µg/mL), polystyrene 440− (100 µg/mL), silver 10− (100 µg/mL), silver 20− (1 µg/mL), endorem (10 µg/mL), ferumoxytol (10 µg/mL), DEAE-dex SPION (1 µg/mL), titanium(IV) oxide (100 µg/mL), silica 50− (100 µg/mL), silica 310− (100 µg/mL), nano SiO_2_ (100 µg/mL) and the positive control menadione (10 µM). Following 24 h incubation (37 °C, 5% CO_2_), the plate was centrifuged at 2000 rpm for 5 min, and the supernatant was removed. Cells were washed in 100 µl HBSS followed by centrifugation, which was repeated twice. During the wash procedure, ThiolTracker Violet dye was prepared to 20 mM in DMSO, and subsequently to the working concentration of 20 µM in HBSS and warmed. One hundred microliters of the dye was added to each well and incubated for 30 min at 37 °C and 5% CO_2_. The plate was centrifuged at 2000 rpm for 5 min, and the supernatant was removed. Cells were washed in 100 µL HBSS followed by centrifugation, and finally suspended in 100 µL of MACSQuant running buffer. Cell suspensions were transferred to a deep well 96-well microplate. Quantification was performed by flow cytometry (MACSQuant) using the FITC channel.

#### 4.2.5. Cell-Free Determination of Influence on Caspase-1

Assessment of the inhibition of caspase-1 was performed using the Caspase-1 Inhibitor Drug Detection Kit following the manufacturer’s protocol. Belnacasan was prepared at a concentration of 10 µM, and polystyrenes were prepared at concentrations of 100 µg/mL in deionized water. Fifty microliters of each was added in triplicate to a 96-well microplate. Active caspase-1 was reconstituted in reaction buffer following the kit protocol, 5 µL of which added to each of these wells.

Assay controls were added to the plate as follows: 50 µL of deionized water as background control, caspase-1 control of 50 µL of deionized water and 5 µL active caspase-1, and positive inhibition control comprised of 50 µL of deionized water, 5 µL active caspase-1 and 1 µL of caspase-1 inhibitor (Z-VAD-FMK, provided with the kit). “Master Mix” was prepared following the assay protocol. Dithiothreitol (DTT) was added to the 2× reaction buffer to a final concentration of 10 mM. To this, 1 mM *N*-Acetyl-Tyr-Val-Ala-Asp-7-amido-4-trifluoromethylcoumarin (YVAD-AFC) substrate, a synthetic peptide substrate cleaved by active caspase-1 to release free 7-Amino-4-trifluoromethylcoumarin (AFC) quantifiable by fluorometry, was added at 10% total volume. Fifty microliters of Master Mix was added to each well and incubated at 37 °C for 1 h.

Fluorescence in each well was measured using a CLARIOstar plate reader with excitation and emission wavelengths of 440 nm and 505 nm, respectively.

Average fluorescence values of background control wells were subtracted from all sample wells. Caspase-1 activity in treated samples was calculated as a percentage of the activity present in negative control wells.

#### 4.2.6. Leukocyte Proliferation Following 48-h Exposure to Nanomaterials

Assessment of leukocyte proliferation was performed as described by Liptrott et al. [56]. Briefly, PBMC suspensions were adjusted to a density of 2.5 × 10^6^ cells/mL and plated at a volume of 100 µL/well in 96-well round-bottomed plates. Conditions consisted of an untreated control, polystyrene 180+ (100 µg/mL), polystyrene 180− (100 µg/mL), polystyrene 275+ (100 µg/mL), polystyrene 300− (100 µg/mL), polystyrene 440+ (100 µg/mL), polystyrene 440− (100 µg/mL), silver 10− (100 µg/mL), endorem (10 µg/mL), ferumoxytol (10 µg/mL), DEAE-dex SPION (1 µg/mL), silica 50− (100 µg/mL), silica 310− (100 µg/mL), nano SiO_2_ (100 µg/mL), and the above treatments in combination with PHA (5 µg/mL final concentration). All conditions were prepared in triplicate. Plates were incubated for a total of 48 h at 37 °C, 5% CO_2_. For the final 16 h, 1 µCi of [3H]-thymidine was added to each well. Cells were harvested onto a filtermat using a Tomtec Harvester 96 and sealed in a sample bag with MeltiLex melt-on scintillator. Incorporated radioactivity was measured on a Perkin-Elmer MicroBeta detector.

#### 4.2.7. Statistical Analysis

Statistical analysis was performed using GraphPad Prism 6 for Windows (GraphPad Software, La Jolla, CA, USA). Statistical differences were determined using either one-way or two-way analysis of variance (ANOVA) where appropriate, and Dunnett’s multiple comparison tests. A *p*-value < 0.05 was considered as statistically significant.

## Abbreviations

AFC	7-Amino-4-trifluoromethylcoumarin
ANOVA	Analysis of variance
DEAE-dex	Diethylaminoethyl-dextran
DMSO	Dimethyl sulfoxide
DTT	Dithiothreitol
FBS	Fetal bovine serum
FITC	Fluorescein isothiocyanate
GM-CSF	Granulocyte-macrophage colony-stimulating factor
HBSS	Hanks’ balanced salt solution
HEPES	4-(2-hydroxyethyl)-1-piperazineethanesulfonic acid
IFN	Interferon
IL	Interleukin
LPS	Lipopolysaccharide
NLRP3	NLR family pyrin domain containing 3
PBMCs	Peripheral blood mononuclear cells
PE	Phycoerythrin
PHA	Phytohemagglutinin-M
REDOX	Oxidation-reduction
ROS	Reactive oxygen species
RPMI-1640	Roswell Park Memorial Institute 1640 medium
SPION	superparamagnetic iron oxide nanoparticle
TNF	Tumor necrosis factor
YVAD-AFC	N-Acetyl-Tyr-Val-Ala-Asp-7-amido-4-trifluoromethylcoumarin
Z-VAD-FMK	Carbobenzoxy-valyl-alanyl-aspartyl-[O-methyl]-fluoromethylketone
